# Hazardous Effects of Pesticides on Human Health

**DOI:** 10.3390/toxics12030186

**Published:** 2024-02-28

**Authors:** Balázs Ádám, Pierluigi Cocco, Lode Godderis

**Affiliations:** 1 Institute of Public Health, College of Medicine and Health Sciences, United Arab Emirates University, Al Ain P.O. Box 15551, United Arab Emirates; 2 Centre for Occupational and Environmental Health, Division of Population Health, University of Manchester, Manchester M13 9PL, UK; pierluigi.cocco@manchester.ac.uk; 3 Centre for Environment and Health, Department of Public Health and Primary Care, Katholieke Universiteit Leuven, 3000 Leuven, Belgium; lode.godderis@kuleuven.be; 4 IDEWE, External Service for Prevention and Protection at Work, 3001 Leuven, Belgium

Pesticides, a major group of biocides, are designed to control harmful and/or unwanted organisms [1]. Pesticides are used in large amounts and play a crucial role not only in agriculture but also in public and veterinary practices to kill or impair the multiplication of weeds, insects, fungi, and other pests. These chemical products are designed to exert toxicity on the target species, but they may also inadvertently pose unintended harmful effects on human health [2]. This Special Issue explores the hazardous effects of pesticides on human health, focusing on the diverse pathways of exposure and the complex toxicological profiles of pesticide formulations.

According to a systematic review, an estimated 385 million people suffer unintentional acute pesticide poisoning globally each year, causing 11,000 deaths; two-thirds of these cases occur in East and South Asia [3]. The number of people who are chronically exposed to low doses is even higher. Pesticide applicators face primarily occupational exposure, but ingredients in pesticide formulations can also reach the general public through various environmental pathways [4]. Although the health effects of pesticides have long been studied and a multitude of toxicological paths of mechanisms have been explored, ongoing research is essential due to the wide and expanding array of pesticides. New ingredients, adjuvants or co-formulants, novel formulant compositions, and complex product structures, such as encapsulated pesticide products, are continuously being developed [5,6]. The chemical industry pursues maximizing agricultural output against the evolving resistance of target organisms and human activity-driven environmental changes.

Climate change and the consequent alterations in the local ecosystems necessitate continuous innovation, including new technologies in pesticide production [6,7]. Climate change is expected to increase the use of pesticides with growing total amounts, higher local doses, increased application frequencies, and a wider scope of varieties of applied pesticide products, which is necessitated by the changing distribution of pests and reduced environmental concentrations of pesticides due to increased volatilization and accelerated degradation as a consequence of increased humidity, elevated temperatures, and direct exposure to sunlight [8,9]. Hence, increased exposure levels, prolonged exposure, and reduced susceptibility to pesticide absorption can be expected in an occupational setting, especially during heat waves [9].

In recent years, numerous studies have repeatedly proven that the toxicological profile of pesticide formulations can differ from their active ingredients [10]. The complex chemical mixtures of product formulations that contain adjuvants in addition to their active ingredient are frequently found to be more toxic than the active ingredient alone. A systematic review by Nagy et al. found that the majority of studies comparing the toxicity of pesticide active ingredients to their product formulations observed an increased toxicity of formulations, which in most cases was attributable to the adjuvants and their interactions [10]. This phenomenon is well exemplified by the debate around the carcinogenicity of the most widely used herbicide, glyphosate. The International Agency for Research on Cancer (IARC) classified glyphosate as a “probable human carcinogen” (group 2A) in 2015 [11], while other reputable international and national organizations did not arrive at the same conclusion at the same time [12,13]. Since then, research has pointed out that the genotoxic and carcinogenic potential of glyphosate-based herbicide formulations can be substantially increased compared to the active ingredient glyphosate alone [14,15,16]. Therefore, in addition to the effects of chronic low-level exposures, such as genotoxicity, carcinogenicity, teratogenicity, sensitization, and neurotoxicity, the intricate interactions of the various components of product formulations demand thorough toxicological assessments to manage human health risks effectively. For these reasons, this Special Issue aimed to collect novel scientific evidence from recent studies investigating the unwanted effects of environmental pesticide exposures on human health.

Detecting early signs of neurotoxicity poses challenges, yet studies demonstrate adverse effects on the nervous system at low pesticide doses. Research by Hirai et al. (contribution 1) identified neurotoxic effects of the neonicotinoid pesticide imidacloprid in mice, revealing behavioral changes and altered neurotransmitter levels. They observed decreased activity in behavior tests and a decrease in the level of monoamine neurotransmitters in the olfactory bulb and the stratum of the brain of test animals.

Another frequently observed harmful effect of pesticides that can be induced by chronic low-dose exposures is sensitization, leading to allergies. Therefore, the detection of the skin sensitization potential of pesticides is crucial to the assessment. Yang et al. (contribution 2) measured luciferase enzyme activity in KeratinoSens cells and LuSens cells using standardized assay models to evaluate the skin sensitization potential of ten and eleven agrochemicals and compared the results to information available on sensitization in animal testing databases. Using the in vitro systems, benomyl, pretilachlor, fluazinam, terbufos, butachlor, and carbosulfan were detected as sensitizers, and glufosinate ammonium, oxiadiazon, tebuconazole, and etofenprox were correctly detected as non-sensitizers. The authors observed conflicting results only for diazinon, which proves the ability of the applied KeratinoSens assay and LuSens assay to test skin sensitization potential with high precision.

Oxidative stress, a pathomechanism often induced by pesticides, leads to various adverse health outcomes. Three articles in this Special Issue investigated oxidative stress caused by pesticides in different contexts. Studies on mancozeb, ipconazole, and glyphosate-based herbicide formulations illustrate the correlation between oxidative stress and liver damage, neurotoxic potential, and cytotoxic effects, respectively. Nuchniyom and her colleagues (contribution 3) could observe an increase in a series of molecular markers of oxidative stress induced by the widely used fungicide mancozeb in orally exposed male Wistar rats, along with a marked hepatotoxic effect measured by markers of liver injury. Both the oxidative stress and the liver damage were effectively prevented by *Nelumbo nucifera* petal tea extract. Another widely used fungicide, ipconazole, was proved by Villaorduña et al. (contribution 4) to increase the production of reactive oxygen species, decrease expression of antioxidant genes, induce inflammation and overexpression of inflammation genes, and reduce cell viability by inducing cell death in SH-SY5Y neuroblastoma cells in vitro. The findings point out the neurotoxic potential of this pesticide. Finally, Makame and her colleagues (contribution 5) investigated oxidative stress and cytotoxicity induced by glyphosate, the most widely applied but currently highly debated herbicide, compared to the same effect of three glyphosate-based herbicide formulations and their other ingredients. They found that glyphosate alone did not significantly affect cell viability, while the formulations and their co-formulant ingredients induced a considerable cytotoxic effect from a quite low concentration that could be explained by increased oxidative stress. The results are in line with several other observations that prove the increased toxicity of pesticide product formulations compared to their active ingredients.

The combined effect of pesticide mixtures was clinically examined by Liang et al. (contribution 6), revealing diverse clinical symptoms and increased risks of acute respiratory failure in patients exposed to methomyl or its mixture with cypermethrin. They analyzed the health outcomes of patients acutely intoxicated with the carbamate insecticide methomyl, the pyrethroid insecticide cypermethrin, or their mixture. The 63 patients treated over a 16-year period developed a wide range of clinical symptoms, and 7 of them died as a consequence of their intoxication. The authors found that exposure to methomyl as well as to the methomyl and cypermethrin pesticide mixture significantly increased the risk of acute respiratory failure, which suggests that methomyl is the major contributor to the toxicity of the pesticide mixture.

The six articles in this Special Issue significantly contribute to our understanding of pesticide toxicity, urging further research on this critical environmental and occupational health concern. The findings underscore the importance of continuous investigation to safeguard human health against the evolving landscape of pesticide use and exposure.
